# Experimental dosimetry of EDR2 films in scanning carbon‐ion irradiation

**DOI:** 10.1002/acm2.13636

**Published:** 2022-05-20

**Authors:** Weiwei Wang, Yu Deng, Zhijie Huang

**Affiliations:** ^1^ Department of Medical Physics Shanghai Proton and Heavy Ion Center Shanghai China; ^2^ Shanghai Key Laboratory of Radiation Oncology(20dz2261000) Shanghai China; ^3^ Shanghai Engineering Research Center of Proton and Heavy Ion Radiation Therapy Shanghai China

**Keywords:** carbon ion, EDR2 film, film dosimetry, pencil beam

## Abstract

**Purpose:**

To investigate the dose‐sensitometric response of extended dose range (EDR2) films to scanning carbon‐ion beams and to evaluate the applications of the obtained response curves to carbon‐ion dose distributions.

**Methods:**

EDR2 films were irradiated by mono‐energetic scanning carbon‐ion beams with different doses to obtain sensitometric curves at different integrated depth doses (DDDs). Six different DDDs were generated by using a proper buildup for each mono‐energetic beam and were used to investigate the energy dependence. The sensitometric curves were obtained by fitting the net optical density (netOD) to dose at different DDDs. The dose difference between the value converted from the netOD and that calculated in the treatment planning system (TPS) was investigated to evaluate the application scope of the sensitometric curve.

**Results:**

Digitizing the EDR2 film with a resolution of 0.36 (72 dpi) provided a good signal‐to‐noise ratio, and the sensitometric curve was linear at all DDDs of clinically relevant incident kinetic energies in the netOD range of 0.02–1.70 for carbon‐ion film dosimetry. The factors used to convert the netOD to absorbed dose were expressed as a linear function of DDDs, with which the depth dose difference between converted and TPS was less than 3% in the proximal area for incident kinetic energies lower than 307.5 MeV/u.

**Conclusion:**

The EDR2 film is a feasible tool for scanning carbon‐ion beam profile measurements by directly evaluating the netOD distribution with proper digitizing resolution and netOD range. By applying the conversion factors, the EDR2 film can also be employed to perform the percentage depth dose consistency checking and linear energy transfer comparison of carbon‐ion lower than 307.5 MeV/u.

## 1 INTRODUCTION

Due to its dosimetric and biological characteristics, scanning carbon‐ion radiotherapy (CIRT) shows great advantages for cancer treatment. To guarantee therapeutic effects and safety, it is important to verify the absorbed dose via a proper quality assurance (QA) procedure.^1^ For the scanning beam, the dose distribution is overlapped by a large number of scanning spots. The absorbed dose is predicted by the treatment planning system (TPS) based on beam shape, beam position and local linear energy transfer.^2^ The beam shape, position and absorbed dose should be checked in routine quality assurance. Two‐dimensional planar detectors, for example, OCTAVIUS^®^ Detector 729^XDR^ and 1500^XDR^ (PTW‐Freiburg, Germany)^3^ or three‐dimensional dosimetric system with 24 ionization chambers are employed for beam profile and dose measurements in CIRT;^4^ however, the minimum water equivalent depth (WED) and poor resolution also limits their applications.

Film dosimetry is attractive for superficial or small‐field planar dose evaluations because of its high resolution and flexibility, making it a good detector for evaluating the dose of superficial volumes using CIRT. Gafchromic^TM^ EBT3 radiochromic film (Ashland, Inc.) has been recently investigated for use with carbon‐ion irradiation.^5^, ^6^, ^7^ Previous studies revealed that exposed films needed 24 hours to obtain a stable optical density for postexposure intensification,^5^ which made them unsuitable if a fast response was needed. Some studies^6^, ^7^ revealed that the EBT3 film is not sensitive for the dose evaluations lower than 0.5 Gy because the response ratio is less than 0.05/Gy at absorbed doses lower than 1.0 Gy against a background OD of 0.40, and additional dose error is injected from non‐linear dose responses which is also energy‐dependent. EDR2 films are quasi‐real‐time detectors with a background OD of 0.18–0.20, which contain very fine monodisperse cubic AgBr microcrystal emulsion layers on both sides of a polyester film. They are widely used in routine X‐ray QA and relative planar dose distribution measurements in intensity‐modulated radiation therapy.^8^, ^9^, ^10^ As described by the vendor, EDR2 films can be directly exposed to X‐rays and have a wide response range of 20–700 cGy, robust processing, and an approximately linear response between the net optical density (netOD) and doses up to about 600 cGy for MV X‐rays. A previous study introduced single‐hit and double‐hit models to describe the sensitometric curve of an XV film and revealed that in addition to the beam quality dependence on megavoltage photons and heavy ions,^11^, ^12^ there was about a 4%–6% dose‐response difference due to processing time delay^13^ but no obvious dose rate dependence.^14^ To digitize the developed films with an optical density higher than 2.0, a DOSIMETRYPRO^®^ Advantage(red) (VIDAR Systems Corporation, U.S.) was used, and settings of 16‐bits and 72 dpi obtained an optimal signal–noise ratio;^15^ however, these studies focused on photon or proton radiotherapy. For CIRT, the linear energy transfer of the beam varies significantly along its path,^2^ which exacerbates the energy dependence of EDR2 films and eventually restricts the use of films in CIRT to those used in photon radiotherapy.

In scanning beam particle therapy, the beam shape, position and percentage depth dose (PDD) are important for dose calculations and quality assurance. The aim of this work was to investigate EDR2 film dosimetry with a scanning carbon‐ion beam under different LETs to obtain the dose‐response curve, including the energy dependence, and apply it for dose distribution evaluation, for example, beam profile, beam position, and depth‐dose distributions.

## 2 MATERIALS AND METHODS

The carbon‐ion was accelerated to a clinically‐relevant kinetic energy range of 86.2–430.1 MeV/u by a synchrotron in the IONTRIS system (Siemens, Germany). A spread Bragg peak by a 3 mm ripple filter (Rifi) was employed for detector irradiation in this work. The beam intensity was modulated from 1.3 × 10^6^/s to 6.5 × 10^7^/s during irradiation according to the planned particle number in the spots of the scanning path. The integrated depth doses (DDDs)^2^ of the scanning carbon‐ion beam in water were simulated by the Monte Carlo Fluka package, which is the absolute dose normalized to the number of incident carbon ions. The DDD data sets were configured in the Siemens treatment planning system (TPS) Syngo^16^ (V13) as a baseline for dose calculations. The EDR2 film dose calibration was performed according to different DDDs. An EDR2 film (CareStream, U.S.) and Solid Water^®^ HE (Sun Nuclear, U.S.) were respectively employed as the detector and buildup phantom in the experiment. To determine the proper buildup thickness to generate different DDDs, the relative stopping power of the buildup phantom to water was measured to be 1.018 by PeakFinder (PTW, Freiburg). The lateral dimensions of the EDR2 film were 25.4 cm × 30.5 cm, and the buildup phantom was 30 cm × 30 cm. Films were sandwiched between the buildup and base phantom, perpendicular to the incident beam. All experiments were repeated with different film batches. After irradiation, a Duolight densitometer (IBA, Germany) was used to create a reference OD step wedge on all films for cross‐calibration. Films were developed by a medical X‐ray processor (PROTEC, Germany) within 1 h after irradiation, scanned with a DosimetryPRO^®^ Advantage scanner (Vidar, Germany) supported by FilmScan (V2.5, PTW, Germany). The bits per pixel scanning parameter was set to 16. All digitized files were analyzed by ImageJ (V1.52, NIH, U.S.). A standard calibration step wedge (RIT, USA) was used to normalize the film output to the standard OD. An Advanced Markus electron chamber (TM34045, PTW, Freiburg) in a chamber holder (29672/U10, PTW, Freiburg) with a Unidos*^webline^* dosemeter (T100021, PTW, Freiburg) was used to measure the absorbed dose in film response calibration.

### 2.1 Film sensitometric curve based on DDDs

Based on the single‐hit model, the sensitometric function is:

(1) $$\mathrm{netOD}=\mathrm{netO}D_{\max}\left(1-e^{-\alpha D}\right),$$

where netOD is the net optical density after irradiation, netOD_max_ is the saturated net optical density, *α* is a coefficient that describes the film sensitivity, and *D* is the irradiated absorbed dose. When *αD*≪1, the function can be simplified to its linear form:

(2) $$\mathrm{netOD}=\mathrm{netO}D_{\max}\cdot \alpha D=\frac{D}{k}.$$

This means that the netOD distribution is approximately equivalent to the dose distribution for a finite dose. Here, *k* is the slope of the EDR2 film's sensitometric curve for the carbon‐ion beam.

According to the baseline in Syngo, the DDD gradient in the distal region of the carbon‐ion beam with a 3 mm Rifi is 26%–45%/mm relative to the maximum over the whole clinically relevant energy range. This means that the dose uncertainty is too large to perform calibration, so all measurements were performed in the proximal regions. To obtain the sensitometric curve, the irradiation field included nine 5 cm × 5 cm square fields with different absorbed doses up to 8.0 Gy. The criterion of film positioning was that it should be located in the Bragg peak or entrance region, and the relative gradient should be less than 5%/mm. The homogeneity in the beam fields defined within the flattened region was:

$$\begin{array}{ccc} \mathrm{Homogeneity} & = & \left(\mathrm{netO}D_{\max}-\mathrm{netO}D_{\min}\right)\\ & & /\left(\mathrm{netO}D_{\max}+\mathrm{netO}D_{\min}\right)\times 100\%. \end{array}$$

This was used for evaluating the quality of irradiated films. The films with a homogeneity of less than 3% were accepted in this investigation.

The DDD of the carbon‐ion beam with 3 mm Rifi was 100.4–985.5 MeV/(g/cm^2^) in the proximal region. To evaluate the film dosimetry dependence on LET, six beam sets with different DDDs were used to perform the experiment, in which films were positioned at the WEDs in Table 1.

**TABLE 1:** Beam parameters of DDDs for film dosimetry experiment

DDD [MeV/(g/cm^2^)]	Initial energy [MeV/u]	WED [mm]	DDD gradient [/mm]
106.5	424.89	7.7	0.50%
310.6	100.07	7.7	2.30%
603.1	248.35	123.4	0.70%
731.9	195.65	81.3	2.10%
839.9	149.96	50.1	3.90%
950.2	100.07	23.1	1.30%

### 2.2 Dose rate dependence

Since the beam intensity was modulated during irradiation in IONTRIS, a dose rate dependence test was also performed in this work. Two beam plans based on different nominal DDDs of 131.2 MeV/(g/cm^2^) and 950.2 MeV/(g/cm^2^) were used to irradiate one film with three different intensities from low (2.40E+06/s), medium (1.70E+07/s), to high (6.50E+07/s). The difference of the netOD at different beam intensities was compared to evaluate the dose rate dependence.

### 2.3 Conversion of netOD to depth dose

**FIGURE 1:**
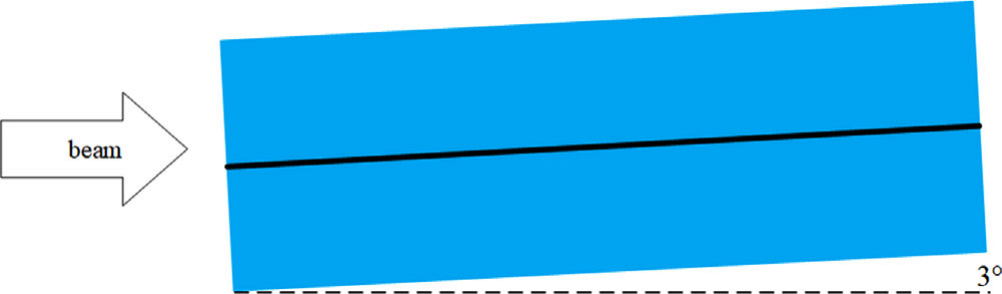
Implemented phantom for depth OD irradiation

One EDR2 film was sandwiched between two 5‐cm‐thick Solid Water phantoms and positioned parallel to the beamline. The couch was pitched 3° to suppress the WED uncertainty,^17^ as shown in Figure 1.

Nine mono‐energetic scanning carbon‐ion beams were used to generate 5 cm × 5 cm homogenous fields to investigate the conversion factors, as shown in Table 2.

**TABLE 2:** Mono‐energies for investigating conversion factors

Energy [MeV/u]	Range in water [cm]
100.07	2.6
248.35	12.6
279.93	15.4
290.71	16.4
301.28	17.4
311.64	18.4
341.69	21.4
361.00	23.4
424.89	30.4

The calibration curve was applied to convert the netOD to the absorbed dose. The depth dose of irradiated energies was exported from Syngo with spacial resolution 1 mm and set as a reference to evaluate the application of conversion factors. The difference between the local dose and Bragg peak width was analyzed. The dose difference index included the average value from the entrance to the distal 50% dose area and 90% dose area around the Bragg peak, while the Bragg peak width index included 90% and 50% dose widths.

## 3 RESULTS

**FIGURE 2:**
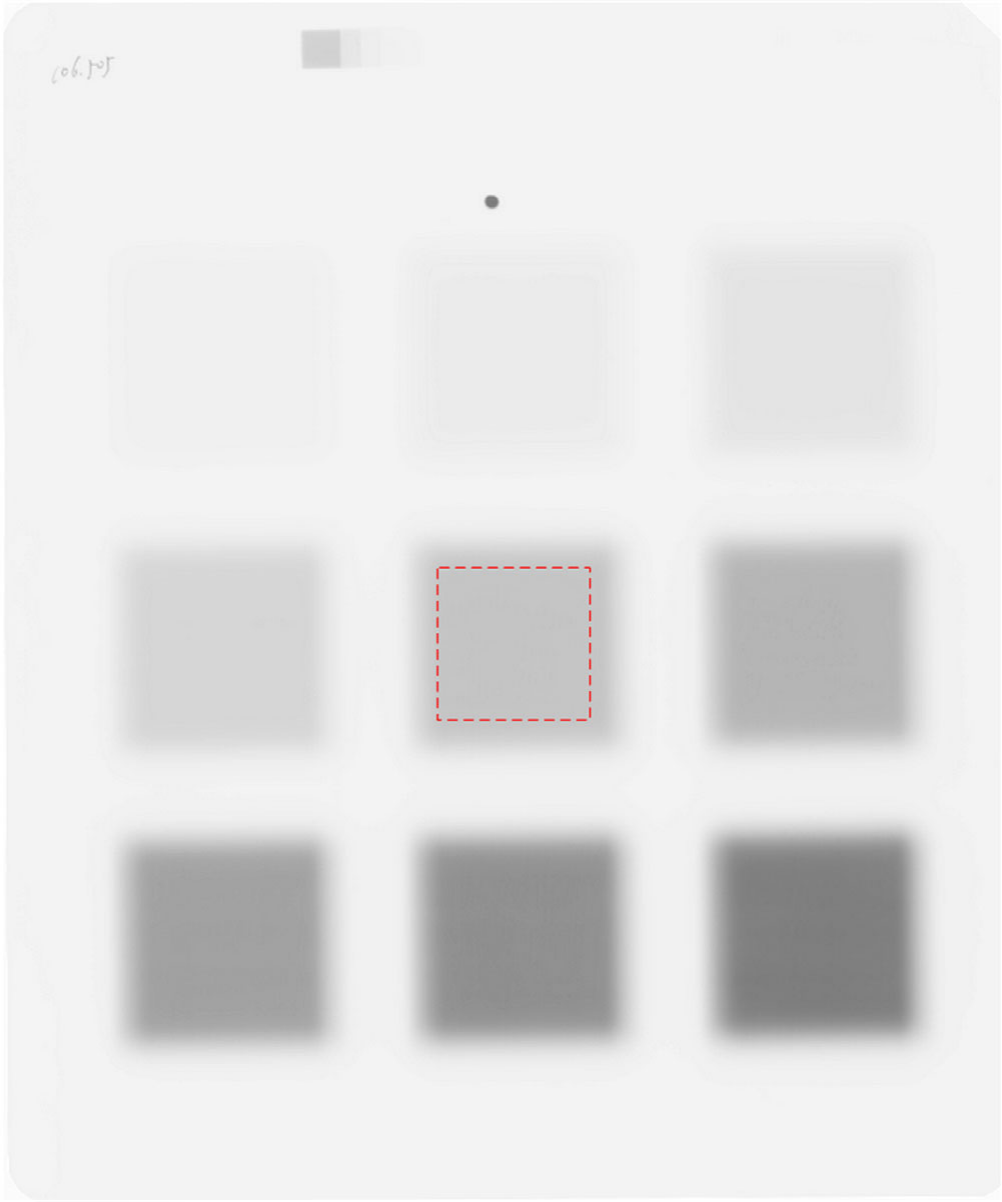
Typical pattern for the sensitometric response investigation of an EDR2 film

A typical pattern for the sensitometric response investigation of the EDR2 film is shown in Figure 2. The flattened region is defined as the full width at half maximum of the irradiated field minus 1.0 cm in this work. The typical value was 1.5%–1.9%.

### 3.1 Sensitometric curve

**FIGURE 3:**
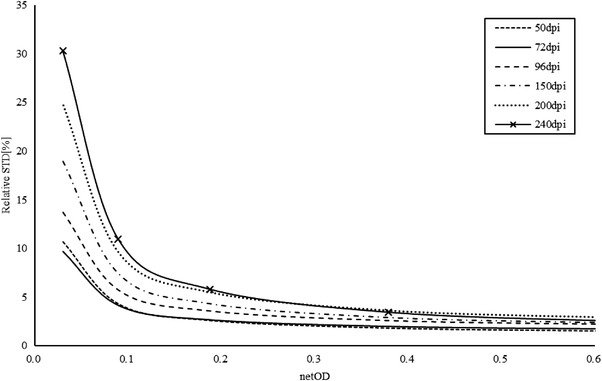
Digitized resolution impact on the netOD reading

A developed film with different netOD was analyzed to study the impact of the scanning resolution. This film was scanned by an EPSON scanner (Expression 11000XL, Japan) with a resolution range of 50–240 dpi. The relative standard deviation (RSTD) is defined as the ratio of the standard deviation and absolute reading. It is used to describe the absorbed dose resolution of the EDR2 film. The relationship between the RSTD and netOD is presented in Figure 3. Three channels (red, green, and blue) were analyzed separately, but only the red channel results are presented since there is only a red channel in the Vidar scanner.

The results revealed that the netOD uncertainty of the digitized EDR2 film was lowest when the scanner resolution was 72 dpi at a low netOD. The difference was less than 3% among the different resolution settings when the netOD was higher than 0.20. So, a resolution of 0.36 was recommended in the Vidar scanner to balance a higher spacing resolution with less uncertainty. The fitting function can be expressed by:

(3) $$\mathrm{RSTD}=\alpha \cdot \mathrm{netO}D^{-1}+\beta ,$$

where *α* = (1.064±0.138) × 10^−3^ and *β* = (6.180±2.049) × 10^−3^.

The uncertainty rapidly increased at a lower netOD, which means a netOD threshold should be set to guarantee the reading reliability. Based on the results, the lower‐limit netOD is 0.02 for the dose resolution criteria of 5%, with which the absorbed dose limit can be calculated based on the dose‐response curve.

**FIGURE 4:**
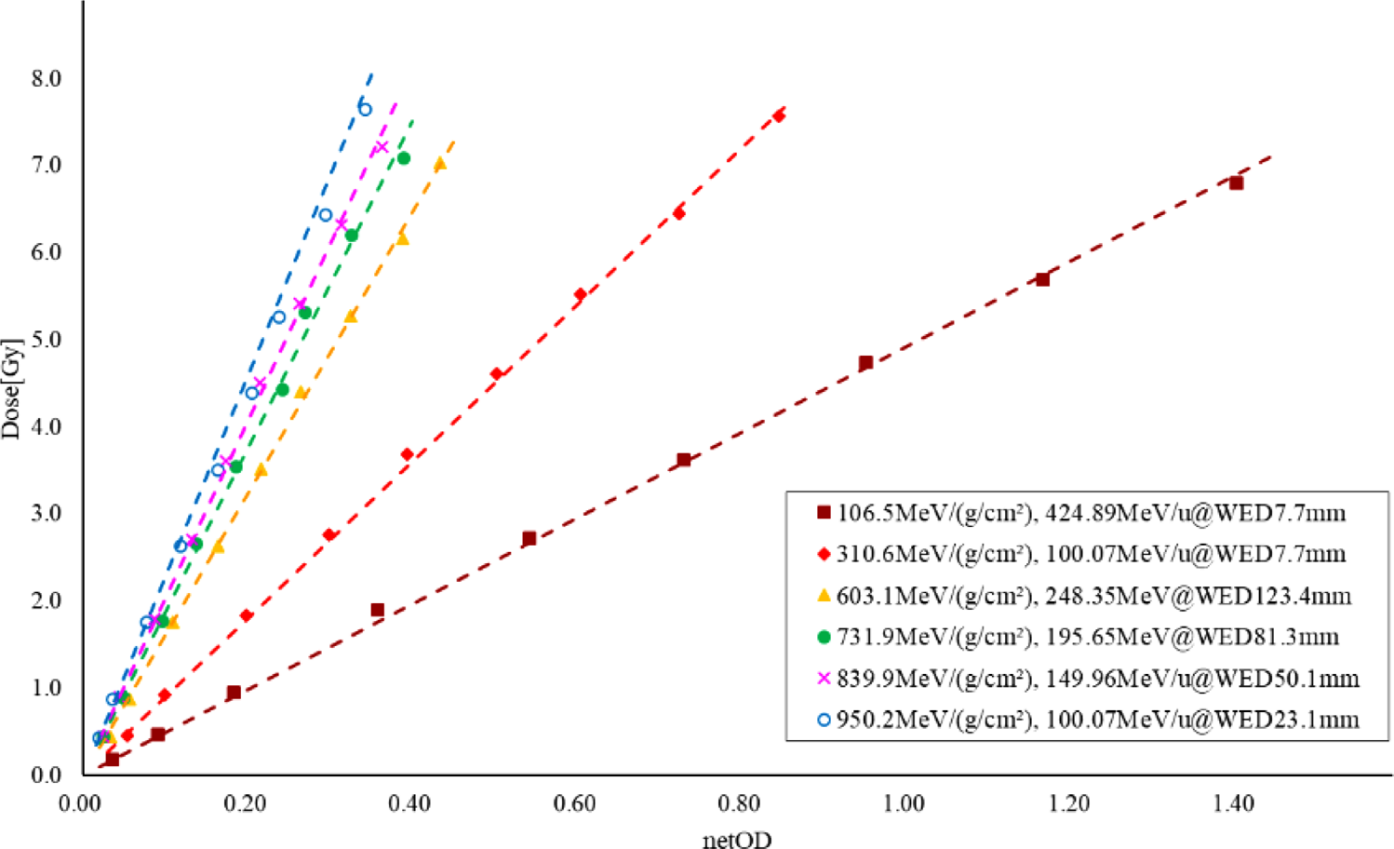
EDR2 film sensitometric curve of carbon‐ion beams with different DDDs

The response curves of the netOD to absorbed dose with different DDDs were analyzed, and a linear function was employed to fit the response curve with a correlation coefficient *R* ^2^ > 0.99 for all sampled nominal DDDs, as shown in Figure 4. The absorbed dose was measured with an Advanced Markus electron chamber. The slopes and dose variations between the fitted and measured values are presented in Table 3.

**TABLE 3:** Sensitometric factors for different DDDs

DDD[MeV/(g/cm2)]	*k*	Dose variation
106.5	4.93±0.18	−3.74%±3.79%
310.6	9.02±0.26	−0.86%±2.90%
603.1	16.20±0.26	0.42%±1.76%
731.9	18.78±0.54	0.71%±3.46%
839.9	20.36±0.38	−0.10%±3.78%
950.2	22.98±0.32	1.18%±3.77%

The sensitometric factor *k* can be fitted by the linear function:

(4) k=a·DDD+b

where *a* = (2.183±0.025) × 10^−2^ g/(MeV·cm^2^) and *b* = 2.424±0.140.

**FIGURE 5:**
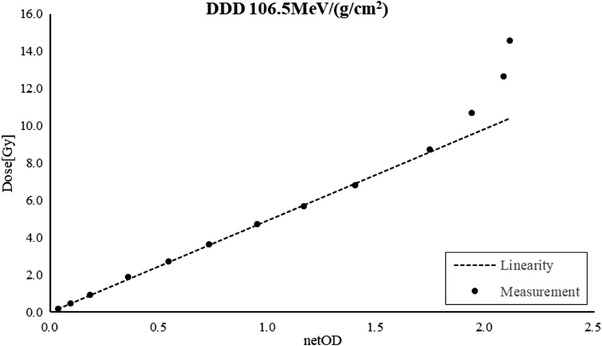
Saturation limit investigation of the EDR2 film

Based on the factor *k* in Table 3, the EDR2 film is more sensitive in the low DDD region, which means a saturation investigation is only necessary for low DDDs. So, a saturation investigation was performed with a DDD of 106.5 MeV/(g/cm^2^). Figure 5 shows the saturated sensitometric curve, which indicates that the netOD upper limit was about 1.70; therefore, the recommended dose range was 0.1–8.4 Gy at a low DDD and 0.5–39.1 Gy at a high DDD for the EDR2 film when used with carbon‐ion irradiation.

### 3.2 Dose rate dependence

The netOD of different beam intensities was sampled and normalized to the lowest values, and the related factors are presented in Table 4. DDD_1_ represents 131.2 MeV/(g/cm^2^), and DDD_2_ represents 950.2 MeV/(g/cm^2^).

**TABLE 4:** Related factors for beam intensity dependence of a scanning carbon‐ion beam

Intensity (PN/s)	Relative netOD	
	DDD_1_	DDD_2_
2.40E+06	1	1
1.70E+07	0.993	1.005
6.50E+07	1.021	1.032

The netOD difference was less than 3.2% among the different beam intensities, which means there is no obvious dose rate dependence for the EDR2 film dosimetry under a scanning carbon‐ion beam.

### 3.3 Conversion of netOD to depth dose

**FIGURE 6:**
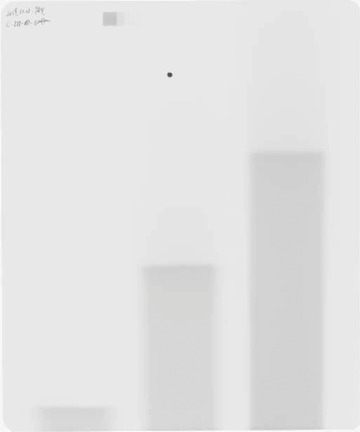
Typical depth‐dose pattern obtained during the investigation of the EDR2 film dosimetry

**FIGURE 7:**
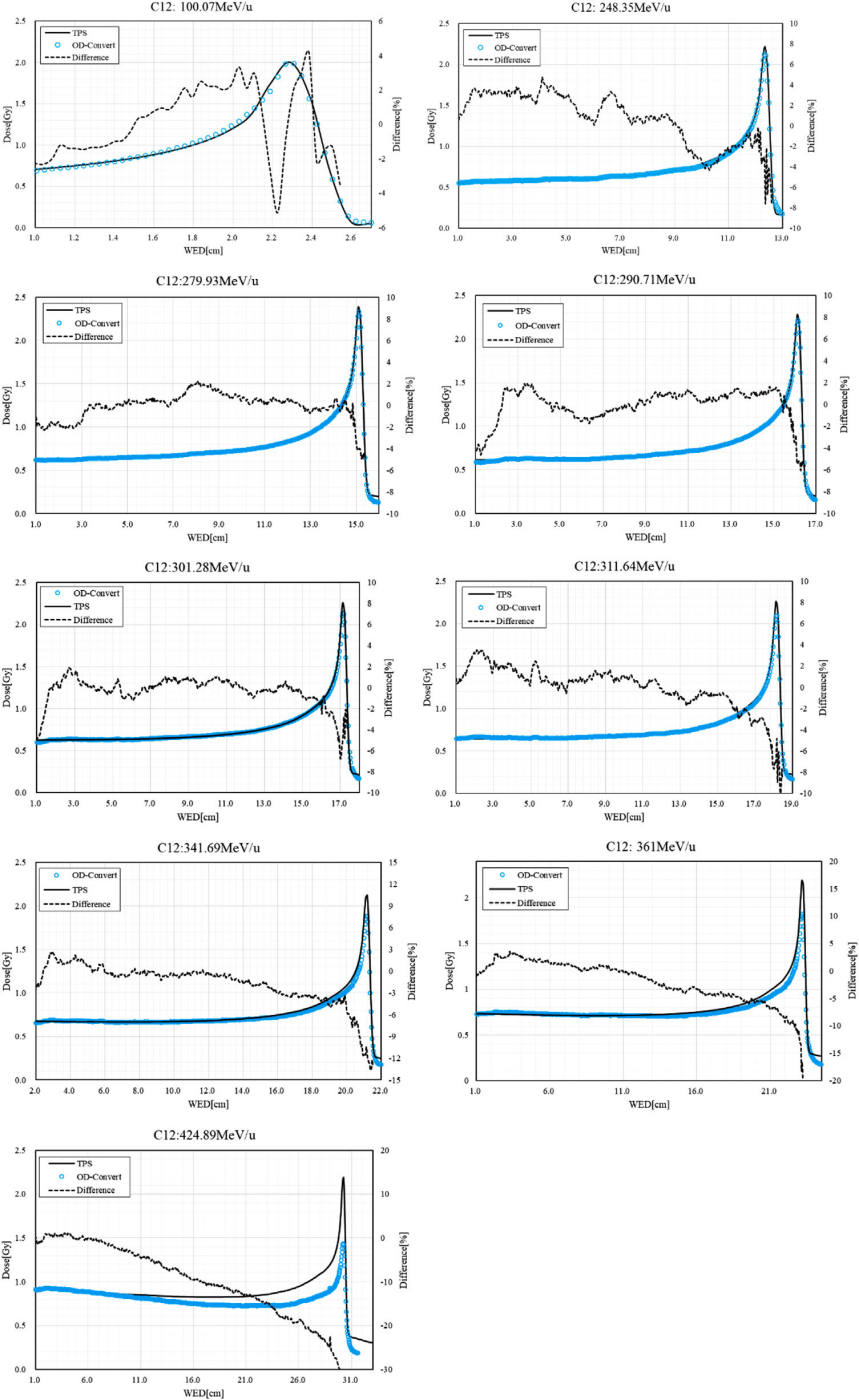
Depth doses converted from the netOD for different scanning carbon‐ion beam energies

The beam pattern for the depth dose investigation is shown in Figure 6. The depth‐dose comparison between the calculation in TPS and netOD‐converted value is shown in Figure 7. The results are presented in Table 5, in which Global represents the entrance to 50% distal area, *R*_90‐90_ and *R*_80‐80_ are the 90% and 80% dose area around the Bragg peak.

**TABLE 5:** EDR2 film application in depth dose evaluation of a carbon‐ion beam

Energy [MeV/u]	Local dose difference		
	Global	*R*_90‐90_	*R*_80‐80_
100.07	−0.09% ± 1.85%	0.14 %± 2.50%	−0.41% ± 2.15%
248.35	0.80% ± 2.48%	−4.26% ± 2.05%	−3.70% ± 2.07%
279.93	−0.09% ± 1.31%	−4.11% ± 0.45%	−3.63% ± 1.09%
290.71	0.76% ± 1.83%	−4.38% ± 1.58%	−3.92% ± 1.65%
301.28	0.65% ± 1.50%	−3.73% ± 0.83%	−3.91% ± 0.82%
311.64	−0.03% ± 1.93%	−6.98% ± 1.26%	−6.91% ± 1.10%
341.69	−1.60% ± 2.63%	−11.41% ± 0.52%	−11.65% ± 0.61%
361.00	−2.12% ± 4.26%	−17.24% ± 1.39%	−17.10% ± 1.25%
424.89	−9.60% ± 8.61%	−33.83% ± 0.97%	−33.41% ± 1.37%

**FIGURE 8:**
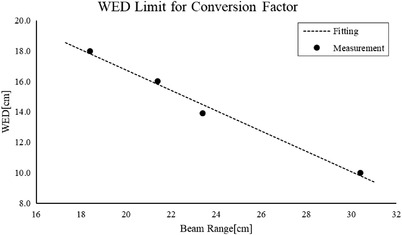
WED limit for the conversion factor

The mean dose difference was less than 3% in the whole range, except for the highest energy, and less than 4.5% in the *R*_90‐90_ area for energies below 301.28 MeV/u. Since the netOD homogeneity at the same DDD was 1.5%–1.9%, the depth doses converted from the netOD by Equation (4) were acceptable in the whole proximal area at energies lower than 301.28 MeV/u, but a WED limit was necessary to use the conversion factor at higher energies. Considering the clinical dose difference criteria is 3%, the WED limits were 18 cm for 311.64 MeV/u, 16 cm for 341.69 MeV/u, 14 cm for 361 MeV/u, and 10 cm for 424.89 MeV/u, as Figure 8 shows. Thus, the maximum WED determined by the nominal beam range is as follows:

(5) WED=−0.6667·range+30.075,WED⩽range.

When the WED equals the range in Equation (5), whose corresponding value was 180 mm, the dose difference was less than 3% in the whole range. So, the energy limit for a dose difference of less than 3% in the whole proximal area was 307.5 MeV/u based on Equation (5).

Consider the lower dose gradient around the Bragg peak for wider peak width with higher incident energy; for example, the gradient at 50% dose in the proximal area is about 4.0%/mm for 100.01 MeV/u versus 0.8%/mm for 361 MeV/u; the Bragg peak shape was also compared by widths of 50%, 60%, 70%, 80%, and 90% of the peak dose, as shown in Table 6.

**TABLE 6:** Bragg peak width difference between measured and reference values

Energy	Peak width difference [mm]				
[MeV/u]	*W*_90‐90_	*W*_80‐80_	*W*_70‐70_	*W*_60‐60_	*W*_50‐50_
100.07	−0.07	−0.17	0.14	0.29	0.33
248.35	0.13	0.05	0.17	0.42	0.70
279.93	−0.14	−0.09	0.03	0.25	0.97
290.71	−0.01	0.03	0.09	0.43	1.56
301.28	0.00	0.03	0.17	0.62	1.38
311.64	0.02	0.02	0.24	0.81	2.01
341.69	0.10	−0.07	0.10	1.26	4.53
361.00	0.05	0.23	0.77	2.29	9.22
424.89	0.17	0.45	1.83	10.36	—

Although the dose difference is over 30% in the *R*_80‐80_ of high energy, the standard deviation of dose difference is less than 2.5% and *W*_80‐80_ is less than 1 mm, which indicate that the Bragg peak profile evaluation with EDR2 film is reliable within the *R*_80‐80_ area for all clinical energy, and *R*_50‐50_ area for energies lower than 279.93 MeV/u.

## 4 DISCUSSION

Since the dose‐response is linear over a wide dynamic range and is especially sensitive to low doses, the EDR2 film is a feasible tool for beam consistency checking in routine quality assurance for scanning carbon‐ion beams. The parameters of mono‐energetic beam measurements in routine quality assurance, for example, beam shape, position, and field homogeneity, can be directly evaluated using the netOD distribution because of their linear response curve. The PDD of mono‐energetic carbon‐ion beam up to 307.5 MeV/u can also be measured with EDR2 films using the energy dependence function of film dosimetry, and the difference between netOD‐converted and TPS is within 3% in the proximal area. The optimal absorbed dose range for such evaluations can be estimated based on the results of this work. Furthermore, the DDD difference between different mono‐energetic carbon ion beam can be evaluated according to the dose response of EDR2 film. In the region after the Bragg peak, the PDD difference increases obviously versus the proximal region. Considering the dose falls off rapidly in the distal region, about 26%–45% per mm, the uncertainty is too high for accurate measurement. Meanwhile, the nuclear inelastic scattering generates more fragments (^+^H, ^2+^He, ^3+^Li, ^4+^Be, ^5+^B) at deeper WED, which makes a different dose‐response in the EDR2 film. So, the dose‐response is changed even in the proximal area of beam of mono‐energies higher than 307.5 MeV/u.

Similar to the study of GafChromic EBT3 film,^7^ the quenching effect of EDR2 film is expressed as a function of LET in this work. With the correction, the proximal PDD converted from netOD agrees with TPS at incident kinetic energy lower than 307.5 MeV/u under local difference criteria 3%, but the performance is not good in the region after the Bragg peak and the proximal region at incident kinetic energy higher than 307.5 MeV/u, which means the quenching effect correction depends on not only the local LET but also the particle components.

## 5 CONCLUSIONS

In this study, EDR2 film dosimetry using a scanning carbon‐ion beam was investigated by digitizing the resolution, netOD, DDD, dose rate, and WED limits. The results indicated that digitizing the film with a resolution of 0.36 (72 dpi) provided a good signal‐to‐noise ratio. A netOD range of 0.02–1.70 is recommended to guarantee both a lower measurement uncertainty and linear sensitometric response. This work proposes a method to convert the netOD values to dose distributions using an appropriate factor *k*. For an incident kinetic energy of less than 307.5 MeV/u, the factor was almost linear with respect to the DDD for the carbon‐ion beam, and the converted dose in proximal region was in fairly good agreement with the dose calculated from Syngo; however this relationship is not guaranteed after the Bragg peak and the proximal region with higher energy than 307.5 MeV/u. No dose rate dependence was observed using the carbon‐ion beam. A WED limit was observed for the conversion factor *k* at high carbon‐ion energies, and this value decreased as the energy increased.

## CONFLICT OF INTEREST

We declare that we do not have any commercial or associative interest that represents a conflict of interest in connection with the work submitted.

## AUTHOR CONTRIBUTIONS

Weiwei Wang: Methodology, investigation, formal analysis, and data curation. Yu Deng: Validation, resources, data curation, and visualization. Zhijie Huang: Conceptualization, writing—original draft, writing—review and editing, and supervision.
